# Differential effects of miR-34c-3p and miR-34c-5p on the proliferation, apoptosis and invasion of glioma cells

**DOI:** 10.3892/ol.2013.1579

**Published:** 2013-09-12

**Authors:** ZHENDONG WU, YUPENG WU, YE TIAN, XIAOFENG SUN, JIXIANG LIU, HONGBO REN, CHAOHUI LIANG, LIGANG SONG, HONGCHAO HU, LIQUN WANG, BAOHUA JIAO

**Affiliations:** 1 Department of Neurosurgery, The Second Hospital of Hebei Medical University, Shi Jiazhuang, Hebei 050000, P.R. China; 2 Department of Neurosurgery, Handan Central Hospital, Handan, Hebei 056000, P.R. China; 3 Department of Neurosurgery, Tianjin Medical University General Hospital, Tianjin 300052, P.R. China

**Keywords:** glioma, miR-34c-3p, miR-34c-5p, proliferation, apoptosis, Notch2

## Abstract

Glioblastoma is the most malignant and common intrinsic brain tumor, but the molecular mechanism of glioma pathophysiology is poorly understood. Recent data have shown that microRNAs regulate the expression of several genes associated with human cancer. In the present study, the function of miR-34c in glioma cells was analyzed. It was demonstrated that miR-34c-3p and miR-34c-5p were downregulated in gliomas, by performing qPCR on tumor tissues from glioma patients and glioma cell lines, compared with normal brain tissues and a normal glial cell line. Furthermore, the miR-34c expression was found to be inversely correlated with glioma WHO grades. Overexpression of miR-34c-3p inhibited U251 and U87 cell proliferation; however, miR-34c-5p only had an effect on U251 cells. Transfection with miR-34c-3p or miR-34c-5p in U251 cells and with miR-34c-3p in U87 cells produced S-phase arrest with G0/G1 reduction and induced cell apoptosis, but no significant changes were observed with miR-34c-5p transfection in U87 cells, normal or negative control groups. However, significant inhibition of glioma cell invasion was observed following transfection with miR-34c-3p and miR-34c-5p. Moreover, it was identified that miR-34c-3p overexpression reduced the expression of Notch pathway members, but miR-34c-5p overexpression did not. Therefore, these results suggest differential tumor suppressor roles for miR-34c-3p and miR-34c-5p and provide new insights into the role of miR-34c in glioma, which includes tumor-suppressing effects on proliferation, apoptosis and invasiveness.

## Introduction

Glioblastoma is the most common and fatal primary tumor in the central nervous system. A combination of surgery, radiotherapy and chemotherapy is widely used to treat gliomas, particularly malignant gliomas. However, the prognosis of patients suffering from malignant glioma remains poor, with a median survival in the range of 12–15 months (1). Thus, the development of efficient treatment therapies that specifically target glioma cells is required.

MicroRNAs (miRNAs) are small, non-coding RNAs that are ~18–24 nucleotides in length and are predicted to regulate the expression of approximately one-third of all human genes at the post-transcriptional and translational levels (2). One miRNA may target several genes, and one gene may be targeted by multiple miRNAs, indicating that miRNAs are involved in the modulation of a wide range of biological processes, such as apoptosis (3). Dysfunction of miRNAs has been reported to commonly occur in several human cancers (4,5), including glioma and its aggressive glioblastoma subtype (6,7). Modulating miRNA activities may provide exciting opportunities for cancer therapy (8).

The miR-34 family (miR-34a, miR-34b and miR-34c), which is described as a p53 effector, has antiproliferative and pro-apoptotic functions (9,10). In particular, downregulated miR-34c is a critical factor that contributes to malignancy in human laryngeal carcinoma, while its overexpression inhibits cell proliferation and induces apoptosis via targeting of c-Met (11). miR-34c has two identified mature miRNAs: miR-34c-3p and miR-34c-5p (12). Although miR-34c-3p and miR-34c-5p have been established as tumor suppressors in a variety of tumors (13,14), their targets and functions in glioma are largely unknown.

In the present study, we analyzed the function of miR-34c-3p and miR-34c-5p in human glioblastoma. U251 and U87 glioblastoma cell lines were transfected with miR-34c-3p or miR-34c-5p mimics. The biological characteristics of U251 and U87 cells were evaluated after enhancing the expression of miR-34c-3p or miR-34c-5p. Additionally, it was investigated whether the effect of miR-34c-3p or miR-34c-5p was associated with the downstream gene Notch2.

## Materials and methods

### Human sample

Formalin-fixed paraffin-embedded human glioma tissue samples (n=18) and normal brain tissues (n=5) were obtained from the Neurosurgery Department of The Second Hospital of Hebei Medical University (Shi Jiazhuang, China). The glioma tissue samples were divided into three groups according to the malignant grade, including Grade II (n=6), Grade III (n=6) and Grade IV (n=6). Data collection and analysis were approved by the ethics committee of Hebei Medical University.

### Cells and cell culture

Human malignant glioma cell lines, U87 and U251, were purchased from the Chinese Academy of Sciences Cell Bank (Shanghai, China). The human normal glial cell line HEB, originally established by Kumar *et al*(15), was purchased from the Guangzhou Institute of Biomedicine and Health, Chinese Academy of Sciences. U251, U87 and HEB cell lines were grown in Dulbecco’s modified Eagle’s medium (DMEM; Hyclone, Logan, UT, USA) enriched with 10% fetal bovine serum (Hyclone) in a 37°C, 5% CO_2_ incubator.

### Transient miRNA transfection

Synthetic, chemically modified miRNA mimics were designed and synthesized by RiboBio (Guangzhou, China). Double-stranded scrambled RNA was used as the negative control (NC). For transfection, 2×10^5^ cells were seeded into each well of 24-well plates and grown for 24 h until they were 30–50% confluent. Cells were washed, placed in Opti-MEM and transfected with oligonucleotides using Lipofectamine™ RNAiMAX Transfection Reagent (Invitrogen Life Technologies, Carlsbad, CA, USA) according to the manufacturer’s instructions. After 4 h, the medium was changed to DMEM/F12 or DMEM-high glucose, respectively, and cells were cultured at 37°C in 5% CO_2_.

### Real-time PCR analysis

Total RNA was extracted using TRIzol (Invitrogen), according to the manufacturer’s instructions. The first strand cDNA was generated by reverse transcription, and then PCR amplification was performed using a real-time PCR cycler (ABI PRISM^®^ 7500 Sequence Detection System; Applied Biosystems, Foster City, CA, USA). Expression of miR-34c-3p and miR-34c-5p were quantified by miR-qRT PCR using SYBR Green RealtimePCR master mix (Toyobo Co., Ltd., Osaka, Japan). U6 was used as an internal control to normalize RNA input. Primers for miR-34c-3p and miR-34c-5p were designed as previously described (14). The results of real-time PCR were analyzed by the ΔΔCT method: ΔCT=CT_selected gene_-CT_U6_, ΔΔCT=ΔCT_therapy group_-ΔCT_control group_, RQ (Relative Quantitation)_therapy group_=2^−ΔΔCT^, RQ_control group_=1.

### MTS assay

The cell survival rate was evaluated using the MTS assay (Promega, Madison, WI, USA). Transfected and control cells in the log phase of growth were seeded in 96-well plates at a cell density of 1×10^4^/well in 100 μl DMEM/F12 or DMEM-high glucose supplemented with 10% fetal bovine serum. For four consecutive days, 10 μl MTS solution was added to each well and mixed, the cells were incubated at 37°C in the 5% CO_2_ incubator for an additional 4 h, and the reaction was stopped by lysing the cells with 200 μl DMSO for 20 min. Absorbance at 490 nm was measured with a Multiskan FC Microplate Photometer (Thermo Fisher Scientific, Logan, UT, USA) and data are expressed as the percentage of the control. All MTS assays were performed in triplicate for each group and experiments were repeated at least twice to confirm the consistency of results.

### Cell-cycle analysis

For cell cycle analysis, transfected and control cells in the log phase of growth were harvested by trypsinization 48 h post-transfection, washed with PBS twice, fixed in 70% ethanol overnight at 4°C and then incubated with 100 μg/ml DNase-free RNase A (Sigma-Aldrich, St. Louis, MO, USA), 0.2% Triton X-100 and stained with 50 μg/ml propidium iodide (PI; Sigma-Aldrich) at 4°C for 30 min. A total of 104 nuclei were examined in a FACSCaliber flow cytometer (BD Biosciences, Franklin Lakes, NJ, USA) and DNA histograms were analyzed by ModFit software (BD Biosciences). Experiments were performed in triplicate.

### Apoptosis assay

Apoptosis analysis was performed in transfected and control cells by staining with the Annexin V-FITC Apoptosis Detection kit (Abcam, Cambridge, MA, USA). In brief, cells were harvested at a density of 1×10^6^ cells/ml in 1X binding buffer and stained with FITC-labeled Annexin V for 15 min at room temperature. Cells were then resuspended in 0.5 ml in 1X binding buffer and stained with 10 μl PI after centrifugation for 5 min at 1,000 × g. Samples were immediately analyzed using the FACSCaliber flow cytometer (BD Biosciences). Data were analyzed by Cell Quest software (BD Bioscience).

### Transwell invasion assay

For the transwell invasion assay, we prepared 8 μm-pore Transwell polycarbonate insert chambers (BD Biosciences) coated with 40 μl Matrigel (BD Biosciences). Following 2 h incubation at 37°C, the Matrigel solidified. Then cells of the transfected and control groups (1×10^5^) in 100 μl of serum-free DMEM/F12 or DMEM-high glucose were added into the upper compartment of the chambers, and 600 μl conditioned medium from U251 and U87 cells was used as the chemoattractant and placed in the bottom chambers. Following 24 h of incubation at 37°C and 5% CO_2_, the medium was removed from the upper chamber. The non-invaded cells on the upper chamber were scraped off with a cotton swab. The invaded cells on the lower membrane were fixed with 4% paraformaldehyde and stained with 0.1% crystal violet (Invitrogen Life Technologies) for 10 min. The number of invaded cells was counted from three randomly selected visual fields, each from the central and peripheral portion of the membrane, using an inverted microscope (CKX41; Olympus, Tokyo, Japan) at ×200 magnification. Each experiment was repeated in triplicate.

### Human Notch2 transcript

The human Notch2 transcript contains a 3751-bp 3′UTR. To explore the possible regulation of Notch2 by miRNAs, *in silico* analysis of miRNAs predicted to target the 3′UTR of its transcript was performed. Several online softwares, including miRanda, TargetScan and PICTAR, predicted that the sequence between nucleotides 2045 and 3073 is likely targeted by miR-34c-3p and miR-34c-5p.

### Western blot analysis

U251 and U87 cells were lysed in 1X cell lysis buffer (R&D systems, Minneapolis, MN, USA) 48 h following transfection. Homogenates were clarified by centrifugation at 14,000 × g for 15 min at 4°C, and protein concentration was measured by NanoDrop (Thermo Scientific). Subsequently, 10% sodium dodecyl sulfate polyacrylamide was used for gel electrophoresis, then gels were transferred to polyvinylidene fluoride membranes (Millipore, Billerica, MA, USA). After permeabilizing and blocking, the membranes were incubated with primary antibody against Notch2 (1:1,000 dilution, Cell Signaling Technology, Inc., Danvers, MA, USA), followed by incubation with HRP-conjugated secondary antibody (1:1,000 dilution, Zymed, South San Francisco, CA, USA). Reactions were developed with ECL or ECL plus (GE Healthcare, Amersham, UK).

### Statistical analysis

SPSS 16.0 software (SPSS, Inc., Chicago, IL, USA) was used for statistical analysis. Spearman’s rank correlation test was used for association analysis between endogenous miR-34c-3p and miR-34c-5p levels and pathological grading. Differences in means were analyzed by the two-tailed t-test. All data are expressed as the mean ± standard deviation. P<0.05 was considered to indicate a statistically significant difference.

## Results

### miR-34c-3p and miR-34c-5p are downregulated in malignant glioma

**Figure 1:**
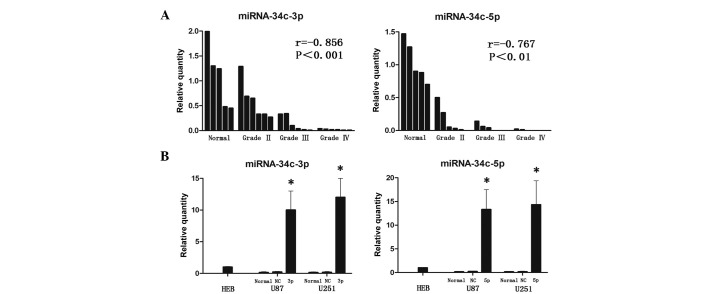
Analysis of the expression level of miR-34c-3p and miR-34c-5p by qPCR. (A) Endogenous level of miR-34c-3p and miR-34c-5p in four grades of formalin-fixed paraffin-embedded human glioma tissue samples. The correlation coefficient (r value) was calculated by Spearman’s rank correlation test. (B) The relative abundance of miR-34c-3p and miR-34c-5p in malignant glioma cell lines, U87 and U251, and the normal glial cell line HEB. ^*^P<0.05 vs. NC group.

The miR-34c-3p and miR-34c-5p expression levels in malignant glioma were measured. All human glioma tissues exhibited lower levels of miR-34c-3p and miR-34c-5p as compared with normal brain tissues, respectively. The level of miRNAs was negatively correlated with the pathological grading of glioblastoma (Fig. 1A). Similarly, U251 and U87 cell lines also exhibited a small amount of endogenous miR-34c-3p or miR-34c-5p (Fig. 1B), indicating an etiological role of miR-34c-3p and miR-34c-5p reduction in glioma progression. Transfection of either pre-miR-34c-3p or pre-miR-34c-5p mimics in U251 and U87 cells significantly increased their respective levels, while transfection with scrambled miRNA negative control (NC) had no effect (Fig. 1B).

### miR-34c-3p inhibits U251 and U87 cell proliferation, but miR-34c-5p only affects U251 cells

**Figure 2:**
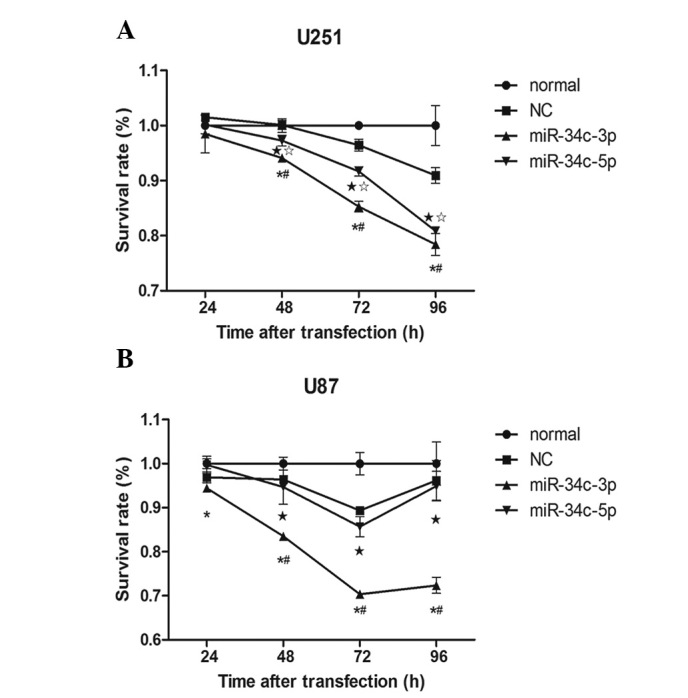
Cellular survival rate following miR-34c-3p or miR-34c-5p mimics transfection was quantified by MTS assay. The survival rate in the normal group is presented as 100%. Data represent the means ± SD of three independent experiments. ^★^miR-34c-5p group versus normal group, P<0.05; ^⋆^miR-34c-5p group versus NC group, P<0.05; ^*^miR-34c-3p group versus normal group, P<0.05; ^#^miR-34c-3p group versus NC group, P<0.05.

Glioblastoma cell proliferation was quantified *in vitro* by MTS assay. Transfection of U251 and U87 cells with miR-34c-3p mimics resulted in 78.42 and 72.37% growth inhibition, compared with normal and NC groups (Fig. 2; P<0.05; 96 h after transfection). However, transfection with miR-34c-5p only inhibited the cell proliferation to 80.84% in U251 cell lines (P<0.05 vs. normal; P<0.05 vs. NC), but not in U87 cell lines (P>0.05 vs. normal; P>0.05 vs. NC). These results implied that miR-34c-3p and miR-34c-5p may function as tumor suppressors in glioma cells *in vitro*. A significant difference in proliferation inhibition was only observed in U87 cells transfected with pre-miR-34c-3p (72.37%) against cells transfected with pre-miR-34c-5p (94.89%), suggesting alternative regulatory pathways are involved (Fig. 2).

### miR-34c-3p and miR-34c-5p overexpression inducesapoptosis in glioma cell lines

**Figure 3:**
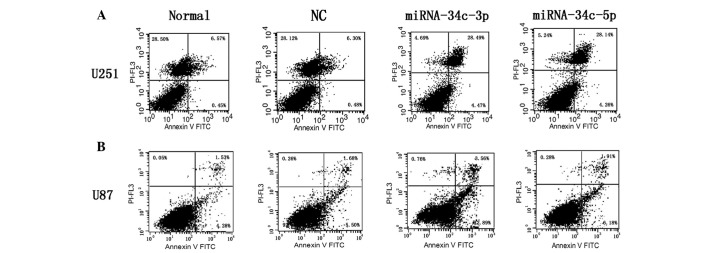
Apoptosis of (A) U251 and (B) U87 cells following miR-34c-3p and miR-34c-5p mimic transfection was analyzed by flow cytometry. Early apoptotic cells are Annexin V+/PI-, late apoptotic cells are Annexin V+/PI+, necrotic cells are Annexin V-/PI+ and healthy cells are Annexin V-/PI-. A representative experiment of the three performed is shown.

To confirm apoptosis following miR-34c-3p or miR-34c-5p overexpression in glioma cell lines, transfected cells were analyzed for Annexin V expression by flow cytometry. Statistically significant increases in Annexin V^+^ and PI^+^ apoptotic cells were observed in miR-34c-3p (28.49%) and miR-34c-5p (28.14%) mimic treatment groups in U251 glioblastoma cells, compared with normal (6.57%) or NC (6.3%) groups (Fig. 3A). However, only miR-34c-3p-treated cells underwent apoptosis in U87 cell lines (3.56%), but no apoptosis was observed in miR-34c-5p-treated U87 (1.91%), normal (1.53%) or NC (1.68%) cells (Fig. 3B).

### Effect of miR-34c-3p and miR-34c-5p on the cell cycle of U251 and U87 cell lines

**Figure 4:**
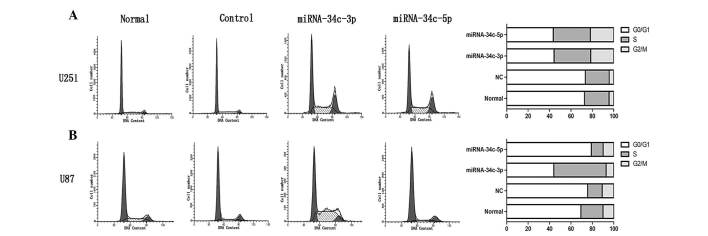
Cell cycle analysis was performed by flow cytometry. Percentages of cells in G0/G1, S and G2/M phases are indicated. Flow cytometry data represented as histograms reveal a significant increase in the percentage of cells in S and G2/M phases in U251 cells following transfection with miR-34c-3p and miR-34c-5p (A), while only S phase arrest in U87 cells after transfection with miRNA-34c-3p (B).

The cell cycle distributions of normal, NC and transfected cells were analyzed by flow cytometry. In U251 cells, as shown in Fig. 4A, the G0/G1 phase fraction of normal and NC cells was 72.7 and 73.44%, respectively. Administration of miR-34c-3p and miR-34c-5p mimics decreased the percentage of cells in G0/G1 phase to 44.24 and 43.58%, respectively. However, the G2/M phase fraction in normal and NC cells were 4.4 and 4.31%, respectively, while miR-34c-3p and miR-34c-5p overexpression increased this fraction to 21.6 and 21.83%, respectively. Similarly, the percentages of cells in S phase in normal and NC cells were lower than those of the transfected cells.

In U87 cells, also shown in Fig. 4B, the G0/G1 phase fraction of normal and NC cells was 69.3 and 75.62%, respectively. While miR-34c-3p overexpression decreased the G0/G1 phase to 44.05%, transfected miR-34c-5p did not affect the G0/G1 phase (79.07%). The S phase fraction of normal and NC cells was 20.66 and 13.61%, respectively, and this increased significantly with miR-34c-3p overexpression to 48.83%; however, this effect did not occur with miR-34c-5p overexpression (11.04%). The G2/M phase fraction showed no significant difference among the four groups.

These results suggested that transfection with miR-34c-3p or miR-34c-5p in U251 cells and with miR-34c-3p in U87 cells produced S phase arrest with G0/G1 reduction. Although, no significant changes were observed with miR-34c-5p transfection in U87 cells or in the normal or NC groups. Cell cycle changes partly explained the differences in cell proliferation, revealing regulatory pathways involving cell division. Notably, miR-34c-3p and miR-34c-5p treatment produced significant levels of apoptosis relative to those of the controls, while no significant sub-G0 population was observed.

### Cell invasive ability is depressed by miR-34c-3p and miR-34c-5p mimic transfection

**Figure 5:**
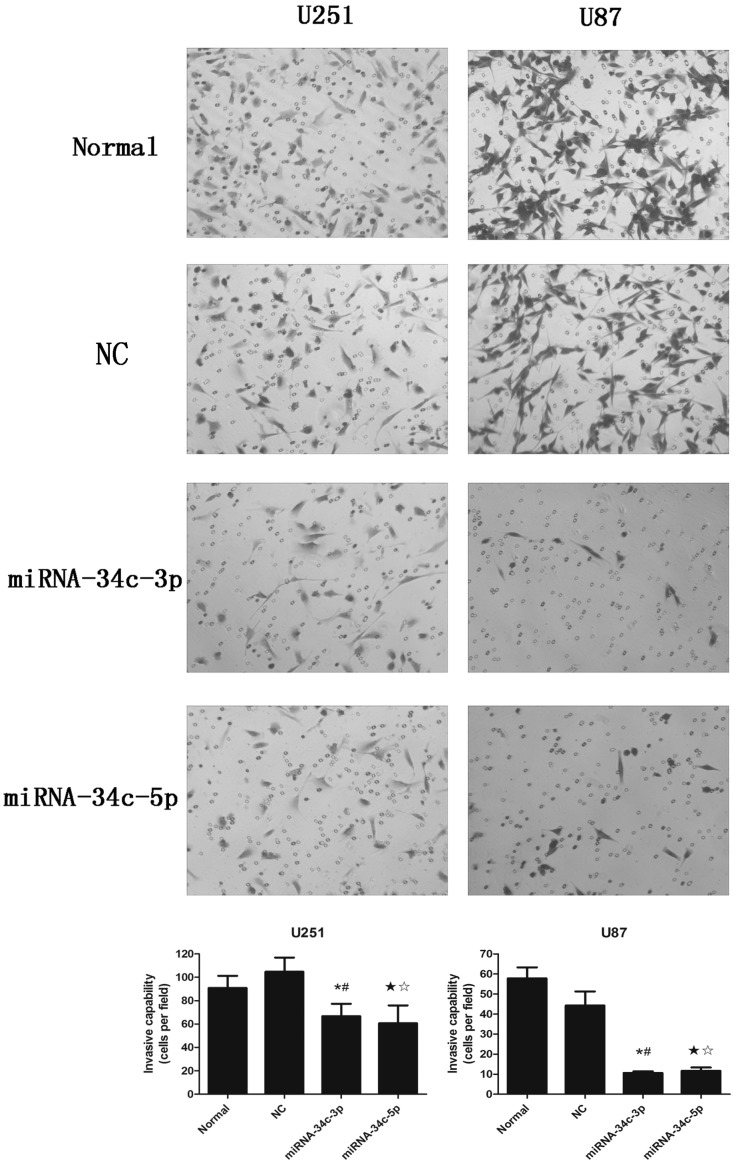
miR-34c-3p or miR-34c-5p mimics impair cellular invasive ability. ^★^P<0.05, miR-34c-5p group versus normal group; ^⋆^ P<0.05, miR-34c-5p group versus NC group; ^*^ P<0.05, miR-34c-3p group versus normal group; ^#^P<0.05, miR-34c-3p group versus NC group.

To evaluate the impact of miR-34c-3p and miR-34c-5p expression on cell invasion, U251 and U87 cells were treated with oligonucleotides and placed on 8 μm-pore size insert chambers coated with a mixture of extracellular matrix proteins. The number of U251 cells invading through the Matrigel following miR-34c-3p and miR-34c-5p mimic treatment was 66.75±10.63 and 60.63±15.32 cells per field respectively, which was lower than that of the normal (90.88±10.38 cells per field) and the NC (104.75±12.06 cells per field) groups (Fig. 5A). Similar results were observed in U87 cells, where invasive activity was decreased to 10.63±2.19 and 11.63±4.75 cells per field in miR-34c-3p and miR-34c-5p mimic-treated cells compared with the normal (57.75±15.55 cells per field) or NC (44.25±19.78 cells per field) cells (Fig. 5B). Our experiments demonstrated that miR-34c-3p or miR-34c-5p overexpression inhibited the invasive ability of U251 and U87 glioblastoma cells *in vitro*.

### miR-34c-3p overexpression reduces expression of Notch2, but miR-34c-5p overexpression does not

**Figure 6:**
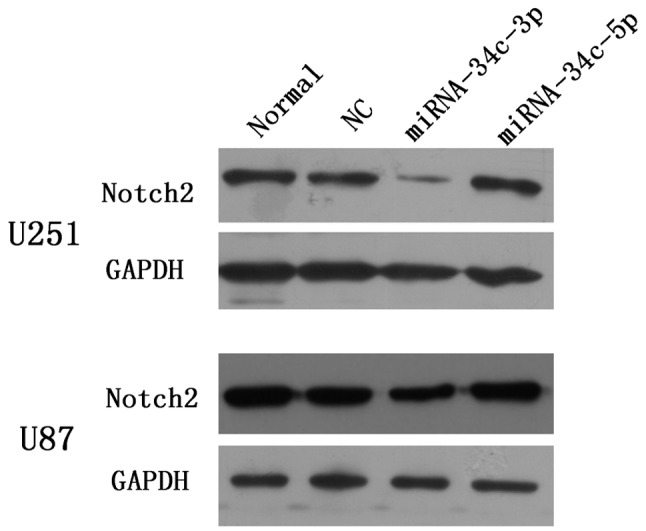
Impact of miR-34c-3p and miR-34c-5p mimics on a component of the Notch signaling pathway. Data are from one of three representative experiments.

miR-34c-3p mimics reduced Notch2 expression by ~15% compared to normal or NC cells (Fig. 6A) in U251 and U87 cell lines. By contrast, Notch2 expression was not downregulated following transfection with miR-34c-5p mimics (Fig. 6B). These data suggested that the tumor suppressor activity of miR-34c-3p in glioblastoma cells may be regulated by the Notch pathway, but that of miR-34c-5p is not.

## Discussion

It is well-documented that the mature miRNA-34 family, as tumor suppressors, shows a global decrease in expression in many different human cancers, including laryngeal carcinoma (11), prostate cancer (16) and cervical carcinoma (17). Although Luan *et al* have demonstrated that miR-34a overexpression inhibited cell migration and invasion in the glioma cell line, U251 (18), the effect of miR-34c on gliomas is unknown.

In the present study, we profiled miR-34c-3p and miR-34c-5p expression in glioblastoma and normal brain tissue. Our data revealed that the level of these two miRNAs was significantly decreased in glioblastoma compared to normal brain tissue. Moreover, the miRNA values were negatively correlated with aggressive behavior of glioblastoma. Overexpression of either miR-34c-3p or miR-34c-5p in U251 cells caused inhibition of proliferation, cell apoptosis, S phase arrest and cell invasion suppression. However, this was not the case in U87 cells. The proliferation inhibition effect of miR-34c-5p was not observed. Furthermore, miR-34c-3p expression clearly produced cell apoptosis and S phase arrest, while no apoptosis or major cell cycle changes were observed with miR-34c-5p. These findings suggested that loss of miR-34c-3p or miR-34c-5p expression may be critical in glioblastoma pathogenesis. miR-34c-3p and miR-34c-5p may target different mRNAs and thus display different results through dissimilar pathways in U87 cells.

Many investigators have demonstrated that expression of the miR-34 family resulted in G0/G1 cell cycle arrest in diverse cellular contexts (19,20). The typical sub-G0 population would occur as well as cell apoptosis. However, the present study showed that expression of miR-34c-3p or miR-34c-5p in U251 and U87 cells caused S phase arrest, indicating that the function of miR-34c was different but complementary to that of miR-34a.

Previous studies have demonstrated that miR-34 family overexpression regulated proliferation and senescence through inhibition of E2F3, BCL-2 and MYC (16,21,22). Transient expression of miR-34a markedly inhibited glioma growth *in vivo* by targeting c-Met and Notch (23). In the present study, we also predicted that Notch2 was the precise intracellular target of miR-34c by using miRanda, TargetScan and PICTAR databases, which was different from that in a previous study (14). Notch signaling is key in the regulation of brain tumor cell proliferation (24). In GBM and astrocytoma, Notch1 and Notch2 are expressed at a high level, while the frequency and the intensity of Notch2 expression is higher than that of Notch1 (25,26). In the present study, it was found that the value of Notch2 was downregulated after transfection with miR-34c-3p, indicating that the effect of miR-34c-3p on cell proliferation inhibition occurred via the Notch2 pathway. However, the level of Notch2 was not significantly different between the miR-34c-5p and control groups. Hence, we hypothesize that the mechanism of miR-34c-5p may be different from that of miR-34c-3p.

To the best of our knowledge, this study provided the first evidence of miR-34c-3p and miR-34c-5p values in glioma patients’ tissues. Transfection with these two miRNAs inhibited the cell proliferation, cell cycle changes, apoptosis and cell invasion. Our findings suggest important roles of miR-34c-3p and miR-34c-5p in glioma etiology and provide potential candidates for treating malignant glioma. More experiments are required to further validate the effect of miR-34c-3p on the Notch2 pathway.
